# Long-Term Cancer Incidence Trends in Korea (2001–2020): An Age–Period–Cohort and Joinpoint Analysis with a Focus on Younger Cohorts

**DOI:** 10.3390/medicina61122179

**Published:** 2025-12-08

**Authors:** Hyungho Lee, Mingyu Kim, Geehyun Song, Jae Young Joung, Hokyung Seo, Jin-Ha Yoon, Jinsoo Chung

**Affiliations:** 1 Department of Urology, National Cancer Center, Goyang 10408, Republic of Korea; 2 Department of Preventive Medicine, Yonsei University College of Medicine, Seoul 03722, Republic of Korea; 3 The Institute for Occupational Health, Yonsei University College of Medicine, Seoul 03722, Republic of Korea

**Keywords:** cancer incidence, young people cancer, annual percent change, age–period–cohort effect

## Abstract

*Background and Objectives*: Cancer incidence patterns in South Korea have shifted markedly over the past two decades, with notable increases among younger generations. Despite growing concern regarding early-onset cancer, comprehensive assessments of long-term age-, period-, and cohort-specific trends across multiple cancer types remain limited. This study examined nationwide cancer incidence trends from 2001 to 2020 using Joinpoint regression and age–period–cohort (APC) modeling. *Materials and Methods*: A population-based analysis was conducted using Korea Central Cancer Registry (KCCR) data, including all primary malignant tumors diagnosed from 2001 to 2020. Incidence rates were calculated by sex and 5-year age groups and standardized to the mid-2000 Korean population. Joinpoint regression estimated annual percent change (APC) and average annual percent change (AAPC), accounting for overdispersion and autocorrelation. Independent temporal effects were evaluated through APC modeling using overlapping 10-year birth cohorts, with the 1961 cohort as the reference. *Results*: Incidence increased for prostate, kidney, breast, and pancreatic cancers, while stomach, liver, lung, and biliary cancers showed continued declines. Colon cancer rose until 2011 and decreased thereafter. More recent birth cohorts exhibited higher risks for prostate, kidney, and pancreatic cancers, whereas older cohorts showed elevated risks for stomach, liver, colon, and biliary cancers. Lung cancer trends diverged by sex, decreasing among men but increasing among women. *Conclusions*: Marked heterogeneity in long-term incidence patterns across cancer types and generations was identified. Rising rates of lifestyle- and obesity-associated cancers in more recent cohorts highlight the need for continued surveillance and targeted prevention strategies. APC-based evaluation provides essential insight into Korea’s evolving cancer landscape and supports future public health planning.

## 1. Introduction

Cancer remains a major cause of morbidity and mortality globally, with rising incidence across both high-income and low-income regions over recent decades [1,2]. These increases are attributed not only to population aging but also to rapid transitions in lifestyle and environmental exposures. In Korea, national cancer statistics have reported more than 220,000 new cancer diagnoses annually, with continued projected increases in both incidence and mortality [3,4,5]. This growing burden is associated with substantial long-term consequences, including psychological distress, diminished physical functioning, and persistent financial hardship among cancer survivors, highlighting the multifaceted nature of cancer as a public health challenge [6,7].

In parallel with these overall trends, a growing body of evidence has documented a rise in cancers occurring in younger adults, often defined as those under 50 years of age. Large-scale population-based studies from the United States and Europe have consistently reported increasing early-onset incidence of colorectal, breast, thyroid, and pancreatic cancers [8,9]. Notably, in the United States, colorectal cancer—historically a disease predominantly affecting older adults—has shown marked increases among individuals in their 20s, 30s, and early 40s, prompting major discussions about screening age modifications [10,11]. Similar patterns have been described in several other Western countries, suggesting that early-onset cancers represent an emerging global epidemiologic shift rather than isolated national phenomena.

Evidence from Asia further supports this trend. Korean and regional studies have reported unique patterns of cancer incidence and survival among adolescents and young adults [12], as well as increasing early-onset incidence for specific tumor types such as breast, gastric, and thyroid cancers [13,14,15,16,17,18]. These findings may reflect broader epidemiologic transitions occurring in countries undergoing rapid socioeconomic development, dietary westernization, and rising obesity prevalence [19,20,21,22,23,24]. Moreover, variations in cancer screening policies, infectious disease patterns, and environmental exposures—such as Helicobacter pylori and hepatitis virus prevalence—can contribute to region-specific incidence profiles that warrant careful examination [25,26,27,28].

Several hypotheses have been put forward to explain the rise in early-onset cancers. Lifestyle-related and metabolic factors, including increased consumption of processed foods, sedentary behavior, and rising obesity rates, have been widely discussed as potential contributors [19,20]. In Korea, obesity prevalence has increased substantially over the past decade [28], and population-attributable fraction analyses have identified obesity and physical inactivity as significant contributors to the national cancer burden [24]. Environmental exposures—including endocrine-disrupting chemicals, air pollution, and hazardous occupational exposures—may also influence cancer risk in younger age groups. Furthermore, earlier and more widespread adoption of cancer screening may influence incidence patterns for cancers such as colorectal cancer [29], though its impact varies across age groups and cancer types.

Despite these internationally recognized trends, research in Korea has largely centered on single cancer types or narrowly defined populations. Several Korean studies have analyzed adolescents and young adults with specific malignancies such as thyroid cancer, gastric cancer, and prostate cancer [13,14,15,16], but comprehensive evaluations across multiple cancers remain rare. Moreover, while age–period–cohort (APC) models are essential tools for disentangling the temporal components underlying cancer trends, APC analyses in Korea have been limited to a small number of cancer types [15]. This gap is significant, given that cohort-related changes often reflect shifts in generational exposures, while period effects can capture the influence of screening, medical improvements, or policy changes.

The Korea Central Cancer Registry (KCCR) provides a unique opportunity to address these gaps. The registry systematically collects nationwide cancer incidence data with standardized reporting practices and is recognized for its completeness and reliability [30]. Leveraging these high-quality data enables robust APC-based analyses across multiple cancer types and allows for detailed characterization of both age-specific and generational trends in the Korean population.

Therefore, this study analyzed long-term cancer incidence trends across 24 cancer types in Korea using age-specific, period-specific, and birth-cohort-specific analyses stratified by sex. By comparing temporal and generational patterns from younger to older age groups, the study aimed to provide a comprehensive understanding of evolving cancer epidemiology in Korea and to identify age- and cohort-related patterns that may inform future public health monitoring.

## 2. Methods

### 2.1. Study Data and Population

This was a nationwide, population-based descriptive epidemiologic study based on cancer registry data. Data on cancer incidence for 2001–2020 were obtained from the Korea Central Cancer Registry (KCCR), which collects information on all newly diagnosed malignant neoplasms in the Korean population through a standardized national reporting system. The KCCR is estimated to cover almost all incident cancer cases in Korea and has been widely used in previous studies of national cancer trends [3,4,5].

We included all primary malignant tumors diagnosed between 1 January 2001 and 31 December 2020 among residents of Korea. Cancer sites were coded according to the International Classification of Diseases, 10th Revision (ICD-10), using the cancer classification dictionary of the Korea Central Cancer Registry. The ICD-10 codes used to define each of the 24 cancer types included in this analysis are listed in Supplementary Table S1.

Thyroid cancer (ICD-10 C73) was excluded from the primary analysis because its incidence trend in Korea is dominated by screening-related overdiagnosis following the widespread adoption of thyroid ultrasonography since the early 2000s. Previous Korean epidemiologic studies have shown that the rapid rise in thyroid cancer incidence reflects detection practices rather than true underlying changes in disease risk [29,31,32]. Including thyroid cancer would therefore obscure cohort- and period-related patterns for other cancer types, which are the main focus of APC modeling in this study.

Incidence rates were calculated by sex and 5-year age groups across the full age range. Age groups were classified in 5-year intervals (e.g., 0–4, 5–9, …, 80–84, and ≥85 years). Age-standardized incidence rates per 100,000 person-years were computed using the mid-2000 Korean resident population as the standard, in accordance with KCCR methodology [3,4,5].

In this study, the term “young people” was used in line with previous literature to refer to individuals younger than 45 years [9,10,11]; however, all statistical analyses were conducted across the entire age range using 5-year age groups. Before conducting trend analyses, we summarized the distribution of case counts and age-specific incidence rates by sex, age group, calendar period, and cancer type to describe the size and structure of the study population.

### 2.2. Statistical Analysis

All analyses were performed separately for males and females. We first examined temporal trends in age-standardized incidence rates using Joinpoint regression. For each cancer type and sex, we modeled the logarithm of the age-standardized incidence rate as a function of calendar year and identified points at which a statistically significant change in trend (joinpoint) occurred. From these models, we obtained the annual percent change (APC) for each line segment and the average annual percent change (AAPC) over the full study period. Statistical significance of APC and AAPC was evaluated using Monte Carlo permutation tests (two-sided, *p* < 0.05), following the method described by Kim et al. [33].

To account for potential overdispersion and autocorrelation in the time-series data, we used the variance and error-structure options provided in the Joinpoint Regression Program (version 4.1.0; US National Cancer Institute, Bethesda, MD, USA). Overdispersion was assessed using the program’s built-in diagnostics, and when overdispersion was present, models were fitted using the Poisson variance with overdispersion option. Autocorrelated errors were allowed when indicated by the model selection criteria recommended in the Joinpoint software (version 5.4.0) documentation and previous methodological work. An age–period–cohort (APC) model was then used to investigate the separate effects of age, calendar period, and birth cohort on cancer incidence [34]. The expected number of cases in age group *i* and period *j* (*E_ij_*) was assumed to follow a Poisson distribution with a log-linear form:

log(*E_ij_*) = log(*P_ij_*) + *μ* + *α_i_* + *β_j_* + *γ_k_* + *ε_ij_*

where *N_ij_* denotes the population size in age group *i* and period *j*, *μ* is the intercept, *α_i_* is the age effect for age group *i*, *β_j_* is the period effect for period *j*, *γ_k_* is the cohort effect for birth cohort *k*, and *ε_ij_* represents the random error term. Birth cohorts were derived from the combination of age group and calendar period. From the fitted APC models, we obtained estimated age, period, and cohort effects, as well as cancer incidence rates by 5-year age groups and birth cohorts.

We constructed overlapping 10-year birth cohorts to ensure stable estimates across early calendar periods with smaller case counts, consistent with established APC methodological recommendations. The 1961 birth cohort was selected as the reference category because it lies near the midpoint of the birth-year distribution of the study population and is commonly used in APC parameterization to enhance interpretability and numerical stability.

All statistical analyses were performed using the Epi package in R (R Foundation for Statistical Computing, Vienna, Austria) for APC modeling and the Joinpoint Regression Program (version 4.1.0) for trend analysis. A two-sided *p* value < 0.05 was considered statistically significant.

## 3. Results

### 3.1. Trends in Age-Adjusted Incidence Rates (APC Analysis)

**Figure 1:**
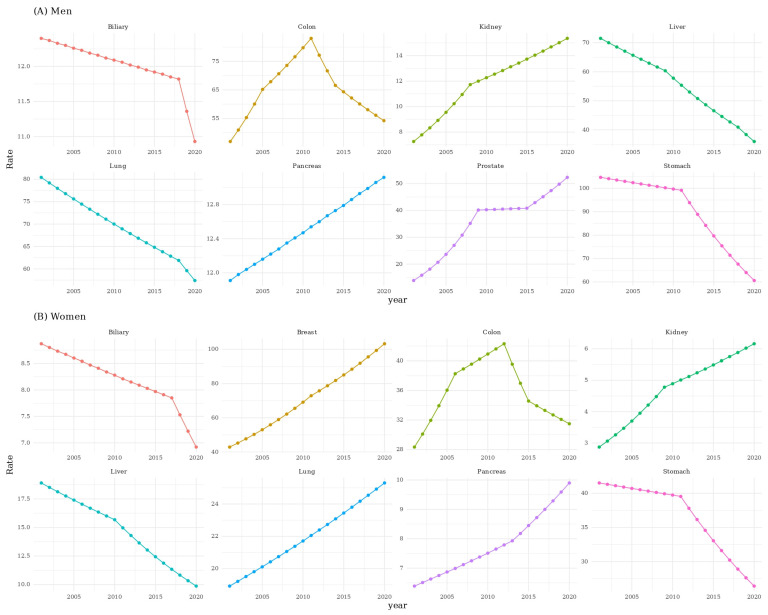
The annual percent change in top 8 cancers stratified by sex.

The APC analysis stratified by sex identified distinct temporal trends across cancers. The age-adjusted incidence rate (AAR) of colorectal cancer demonstrated a reverse U-shaped pattern, increasing until approximately 2011 and decreasing thereafter (AAPC 0.763; 95% CI 0.556–1.101; *p* < 0.05). In males, prostate, pancreatic, and kidney cancers showed increasing AARs (prostate AAPC 7.222; 95% CI 6.687–8.018; *p* < 0.05; pancreatic AAPC 0.511; 95% CI 0.296–0.768; *p* < 0.05; kidney AAPC 4.022; 95% CI 3.711–4.483; *p* < 0.05). In females, breast cancer demonstrated an increasing trend (AAPC 4.729; 95% CI 4.329–5.337; *p* < 0.05), and pancreatic and kidney cancers also increased. Infection-associated cancers declined in males, including stomach (AAPC −2.834; 95% CI −3.139 to −2.516; *p* < 0.05), liver (AAPC −3.550; 95% CI −3.684 to −3.391; *p* < 0.05), and biliary tract cancers (AAPC −0.665; 95% CI −0.875 to −0.322; *p* < 0.05). Lung cancer showed opposite trends by sex, decreasing in males (AAPC −1.758; 95% CI −1.999 to −1.324; *p* < 0.05) and increasing in females (AAPC 1.542; 95% CI 1.293–1.834; *p* < 0.05) (Figure 1).

### 3.2. Age–Period–Cohort Decomposition

**Figure 2:**
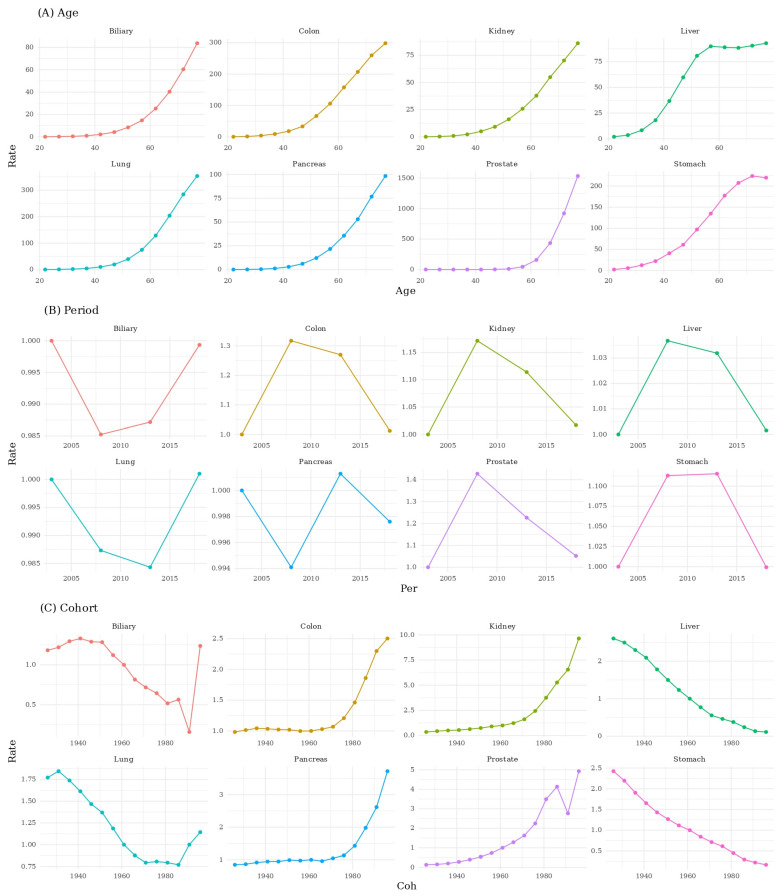
The Age–Period–Cohort effect of top 8 cancers in males.

Age–period–cohort decomposition demonstrated increasing age effects across all cancer types, with higher incidence at older ages. Period effects corresponded to the temporal patterns identified in APC analysis. Period slopes decreased for stomach, liver, and biliary cancers, whereas prostate, breast, pancreatic, and kidney cancers showed increasing period effects. Cohort effects differed across cancers. Successively younger birth cohorts demonstrated lower incidence for stomach, liver, and biliary cancers. In contrast, prostate, pancreatic, kidney, and breast cancers showed higher incidence in more recent cohorts. Colorectal cancer exhibited elevated incidence among younger cohorts during mid-birth cohort periods (Figure 2).

### 3.3. Birth Cohort Effects by Sex

**Figure 3:**
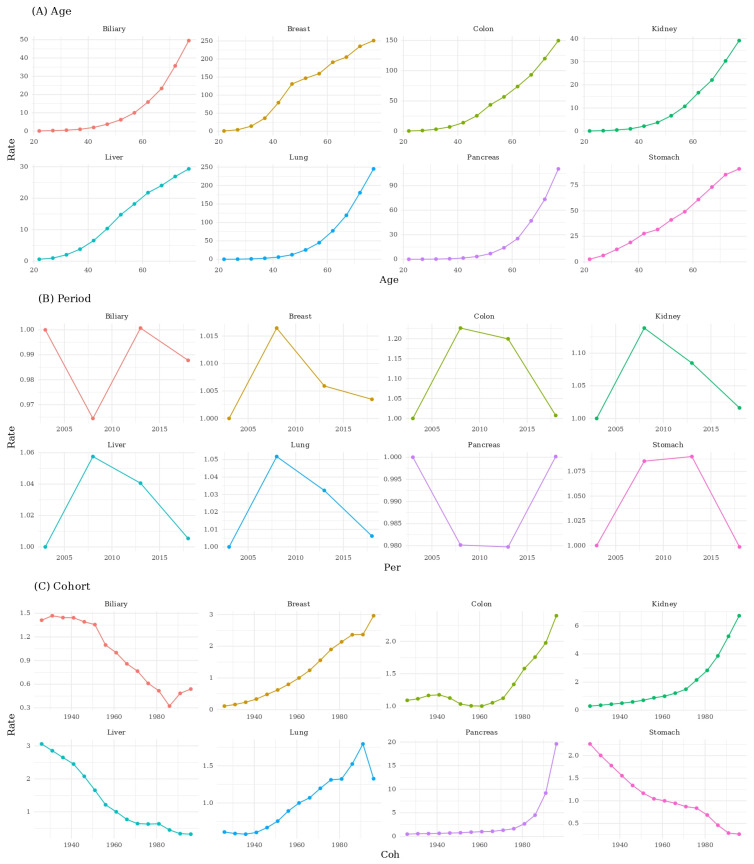
The Age–Period–Cohort effect of top 8 cancers in females.

Sex-stratified cohort patterns varied across cancer types. For stomach, liver, and biliary cancers, younger birth cohorts demonstrated lower incidence than older cohorts in both sexes, with a steeper decrease observed in males. For prostate and kidney cancers in males and breast and kidney cancers in females, incidence increased in younger birth cohorts compared with older cohorts. Lung cancer showed opposite cohort trends by sex. Younger male cohorts demonstrated lower incidence than older male cohorts, whereas younger female cohorts demonstrated higher incidence than older female cohorts (Figure 3).

### 3.4. Age-Specific Cohort Comparisons

**Figure 4:**
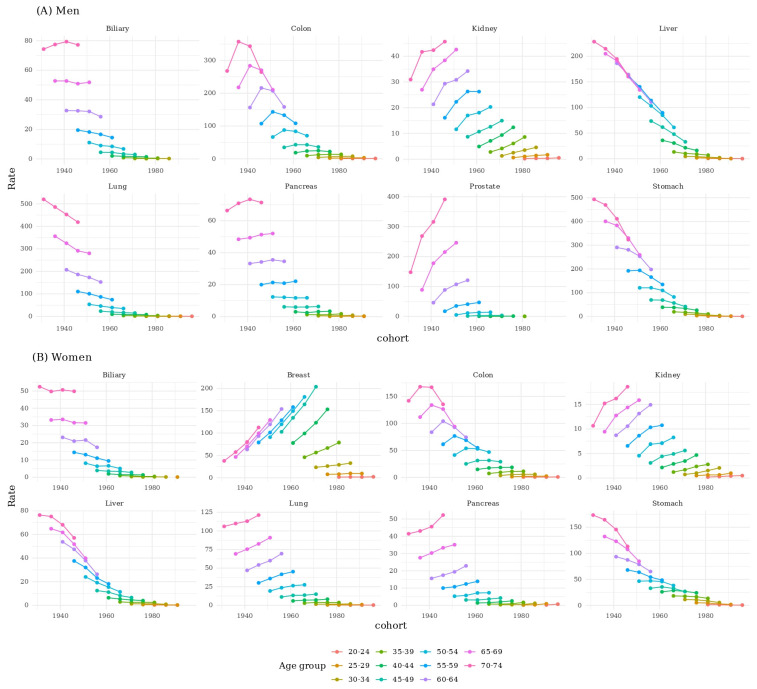
The incidence of top 8 cancers of age groups by birth cohort stratified by sex.

Age-specific analyses showed consistent cohort differences within identical age strata. For stomach, liver, and biliary cancers, older birth cohorts exhibited higher incidence across all evaluated age groups. For prostate, pancreatic, kidney, and breast cancers, incidence was higher in younger cohorts than in older cohorts across age groups ranging from 20 to 24 to 45–49 years. For colorectal cancer, younger cohorts showed higher incidence than older cohorts between ages 20–24 and 45–49 years, while differences were less apparent in older age ranges. For lung cancer, younger male cohorts had lower incidence than older male cohorts across most age groups, whereas younger female cohorts had higher incidence than older female cohorts (Figure 4).

## 4. Discussion

Patterns of cancer incidence in Korea have undergone substantial changes over the past two decades, and the present analysis illustrates how these shifts differ across cancer types, age groups, and birth cohorts. Distinct temporal patterns were observed: prostate, kidney, breast, and pancreatic cancers exhibited sustained increases, whereas stomach, liver, lung, and biliary cancers continued to decline. Colon cancer followed an inflection pattern, rising until approximately 2011 and gradually decreasing thereafter. These contrasting trajectories highlight the heterogeneous nature of Korea’s epidemiologic transition and underscore the importance of evaluating long-term trends from both period and cohort perspectives.

The birth cohort findings further emphasize generational differences in cancer burden. More recent cohorts demonstrated higher incidence for prostate, kidney, and pancreatic cancers, while older cohorts showed greater risks for stomach, liver, biliary, and colon cancers. These cohort-specific patterns are broadly consistent with literature suggesting that exposures accumulated earlier in life—such as diet, obesity, physical inactivity, and other metabolic factors—may have long-term implications for cancer development [19,20,21]. Korea has experienced substantial increases in obesity prevalence and westernized dietary patterns over the past several decades [23,24], and these trends may partly align with the rising incidence of obesity-related cancers. Experimental and mechanistic studies have shown that excess body weight and obesogenic diets can promote tumorigenesis [22], lending biological plausibility to these patterns, although individual-level exposures were not assessed in this study.

National health survey data indicate that obesity prevalence among Korean adults has increased over time, as shown in serial KNHANES analyses [35,36]. Although individual-level exposure data were not available in this study, these population-level trends provide contextual background that may help interpret the rise in several obesity-related cancers among more recent birth cohorts.

Declining trends in stomach, liver, and biliary cancers observed among younger cohorts coincide with improvements in infectious disease control in Korea. The prevalence of Helicobacter pylori has decreased notably following the adoption of clarithromycin-based eradication regimens and updated treatment guidelines [26]. Liver cancer reductions parallel the dramatic decline in hepatitis B virus infection after the implementation of national immunization programs beginning in the 1980s [27,28]. Similarly, Clonorchis sinensis infection has declined due to public health measures, and increasing use of cholecystectomy may have contributed to the downward trend in biliary tract cancers [37,38]. Although these factors may help explain population-level declines, further investigation is needed to clarify their relative contributions.

Lung cancer trends demonstrated a clear divergence between men and women. Male incidence declined, aligning with long-term reductions in smoking prevalence, whereas female incidence increased in accordance with rising smoking rates and targeted tobacco marketing campaigns beginning in the late 1980s [39,40]. Occupational and environmental exposures may also contribute to these sex-specific patterns [40], and future research incorporating exposure histories would be valuable.

This study has several strengths. It uses high-quality nationwide cancer registry data that capture nearly the entire Korean population and applies age–period–cohort modeling to disentangle temporal trends that are not apparent from age-specific analysis alone. The inclusion of multiple cancer types provides a broad perspective on Korea’s epidemiologic transition and highlights distinct generational patterns that may warrant focused attention.

However, several limitations should be noted. The APC framework is subject to inherent identifiability constraints, and although model fit was acceptable, independent interpretation of age, period, and cohort effects requires caution [34]. Second, the registry-based design precludes analysis of individual-level exposures such as lifestyle, environmental determinants, or socioeconomic factors. As a result, potential explanations for observed trends remain speculative. Third, early incidence data may be less complete; restricting the analysis to 2001 onward helped mitigate this issue. Finally, as an ecological study, causal inference cannot be established.

In summary, this study characterizes long-term cancer incidence patterns in Korea across multiple cancer types and reveals substantial heterogeneity in age, period, and cohort effects. These findings provide foundational epidemiologic insight into generational shifts in cancer burden but should be interpreted within the limitations of registry-based data. Continued surveillance and future studies incorporating individual-level risk factors are needed to better understand the drivers of Korea’s changing cancer landscape.

## Data Availability

The cancer incidence data analyzed in this study were obtained from the Korea Central Cancer Registry (KCCR), a nationwide population-based registry administered by the Ministry of Health and Welfare and the National Cancer Center, Korea. Due to legal and regulatory restrictions regarding the protection of personal information under Korean law, the original dataset is not publicly available. Researchers may request access to KCCR data through the official data request process managed by the Korea Central Cancer Registry. This study used de-identified, registry-level data collected for public health and administrative purposes. In accordance with Korean bioethics regulations, studies utilizing such non-identifiable secondary data are typically exempt from institutional review board (IRB) review and do not require individual informed consent. Access to the dataset for this study was conducted under these permissible regulatory conditions.

## Supplementary Materials

The following supporting information can be downloaded at: https://www.mdpi.com/article/10.3390/medicina61122179/s1.
