# Supplementary material for: Long-Term Cancer Incidence Trends in Korea (2001–2020): An Age–Period–Cohort and Joinpoint Analysis with a Focus on Younger Cohorts
Source: Medicina (Kaunas). 2025 Dec 8;61(12):2179. doi: 10.3390/medicina61122179 (PMC12734899; doi:10.3390/medicina61122179)
Supplement: Supplementary file 1 [file medicina-61-02179-s001.zip › STROBE Checklist.pdf]

STROBE Checklist for Population-Based Descriptive Epidemiologic Study

Item No.	Recommendation	Reported in Manuscript
**Title & Abstract**		
**1**	Indicate study design in title/abstract and provide informative summary.	Yes – Title specifies nationwide APC & Joinpoint analysis; abstract summarizes objectives, methods, results, conclusions.
**Introduction**		
**2**	Explain scientific background and rationale.	Yes – Detailed global/Korean trends, early-onset cancer literature, knowledge gap stated.
**3**	State specific objectives and hypotheses.	Yes – Aim clearly defined at end of Introduction.
**Methods**		
**4**	Present key study design elements early.	Yes – Nationwide population-based descriptive epidemiologic study stated at beginning of Methods.
**5**	Describe setting, locations, and time periods.	Yes – KCCR, years 2001–2020.
**6**	Describe eligibility criteria and data sources.	Yes – All malignant tumors; KCCR registry.
**7**	Clearly define variables and coding.	Yes – ICD-10 codes and 24 cancer types; supplementary table provided.
**8**	For each variable, provide data handling and measurement methods.	Yes – Age standardized incidence, 5-year age groups, mid-2000 standard population.
**9**	Describe efforts to address bias.	Yes – Exclusion of thyroid cancer due to overdiagnosis; Describe completeness of KCCR.
**10**	Explain study size and sample description.	Yes – Nationwide dataset; descriptive statistics summarized prior to modeling.
**11**	Explain statistical methods, including: APC/ AAPC estimation, joinpoint modeling, overdispersion, autocorrelation adjustments, APC modeling, cohort construction.	Yes – Fully described in Statistical Analysis section.
**12**	Explain handling of missing data.	Not applicable – Registry completeness >98%; no missing-case handling required.

**Results**		
**13**	Report participant numbers and data flow.	Yes – All cases 2001–2020 included.
**14**	Provide descriptive data (age, sex, cancer type, trends).	Yes – Numeric results and trends provided; figures 1–4 present incidence patterns.
**15**	Report outcome data.	Yes – AAR, APC, AAPC trends, cohort effects.
**16**	Main results with confidence intervals and p-values.	Yes – APC/AAPC with 95% CIs and p-values.
**Discussion**		
**17**	Summarize key findings with reference to study objectives.	Yes – Opening of Discussion.
**18**	Discuss limitations, including statistical modeling constraints, ecological design, absence of individual-level exposure data.	Yes – Explicitly stated.
**19**	Provide balanced interpretation considering aims, limitations, and comparison with literature.	Yes – Supported by references to infectious disease control, obesity, smoking, etc.
**20**	Discuss generalizability (external validity).	Yes – Mentioned registry-wide representativeness of Korean population.
**Other Information**		
**21**	Disclose funding sources and roles.	Yes – National Cancer Center grant noted.
**22**	Ethical considerations / IRB statement.	Yes – Exemption from IRB due to de-identified KCCR data.
