# Supplementary material for: Long-Term Cancer Incidence Trends in Korea (2001–2020): An Age–Period–Cohort and Joinpoint Analysis with a Focus on Younger Cohorts
Source: Medicina (Kaunas). 2025 Dec 8;61(12):2179. doi: 10.3390/medicina61122179 (PMC12734899; doi:10.3390/medicina61122179)
Supplement: Supplementary file 1 [file medicina-61-02179-s001.zip › Supplementary Table S1.pdf]

Supplementary Table S1. List of Selected Cancer Codes According to the International Classification of Diseases, 10th Revision (ICD-10)

Cancer	ICD-10 Code
Lip-oral cavity and Pharynx	C00-C14
Esophagus	C15
Stomach	C16
Colon and Rectum	C18-C20
Liver	C22
Biliary	C23-C24
Pancreas	C25
Larynx	C32
Lung	C33-C34
Breast	C50
Cervix uteri	C53
Corpus uteri	C54
Ovary	C56
Prostate	C61
Testis	C62
Kidney	C64
Bladder	C67
Brain and CNS	C70-C72
Thyroid	C73
Hodgkin lymphoma	C81
Non-Hodgkin lymphoma	C82-C86, C96
Multiple myeloma	C90
Leukemia	C91-C95
Others	Re. C00-C96
