# Supplementary figures and images for: Long-Term Cancer Incidence Trends in Korea (2001–2020): An Age–Period–Cohort and Joinpoint Analysis with a Focus on Younger Cohorts
Source: Medicina (Kaunas). 2025 Dec 8;61(12):2179. doi: 10.3390/medicina61122179 (PMC12734899; doi:10.3390/medicina61122179)

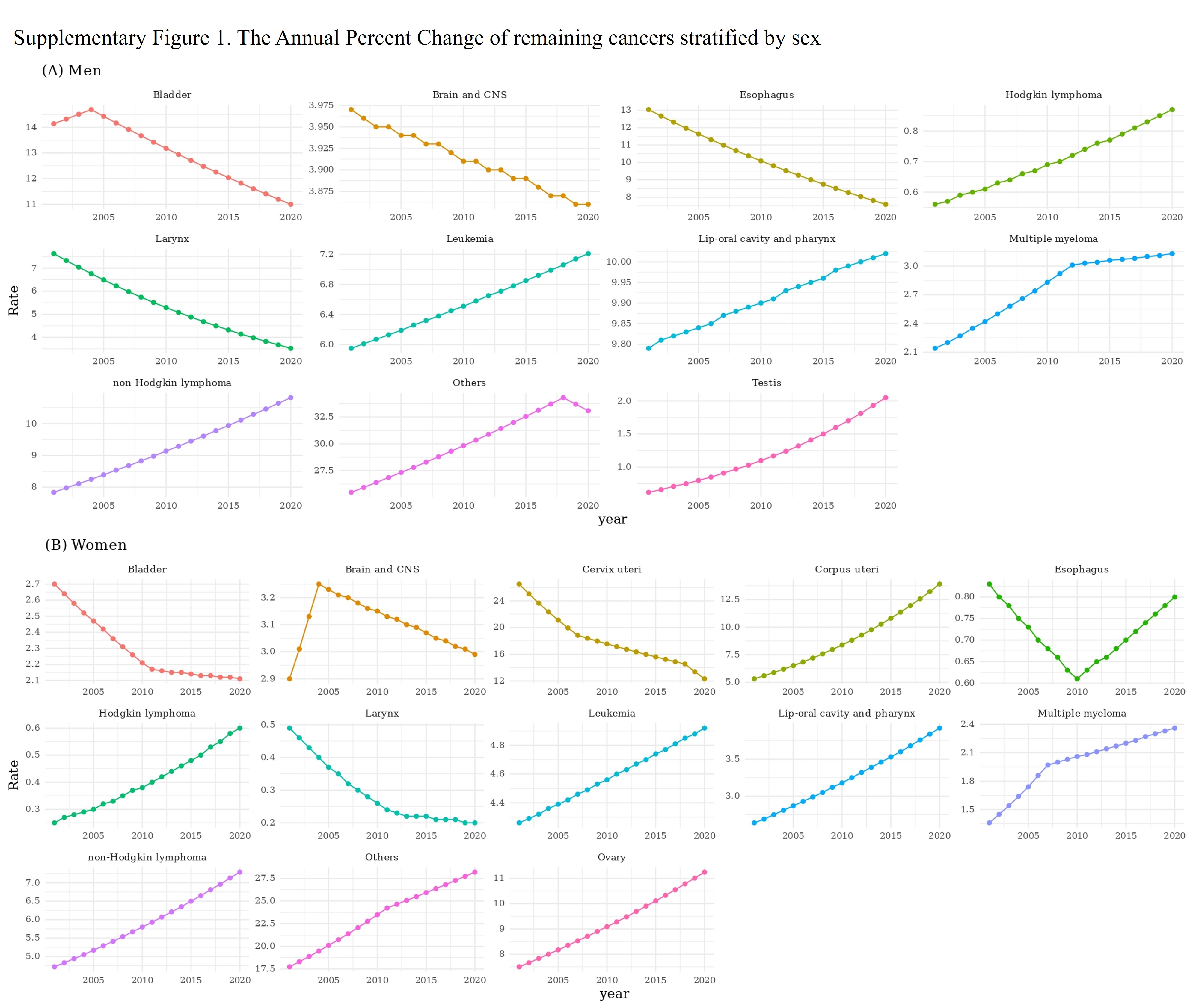

Supplement: Supplementary file 1 [file medicina-61-02179-s001.zip › Figure S1.jpg]

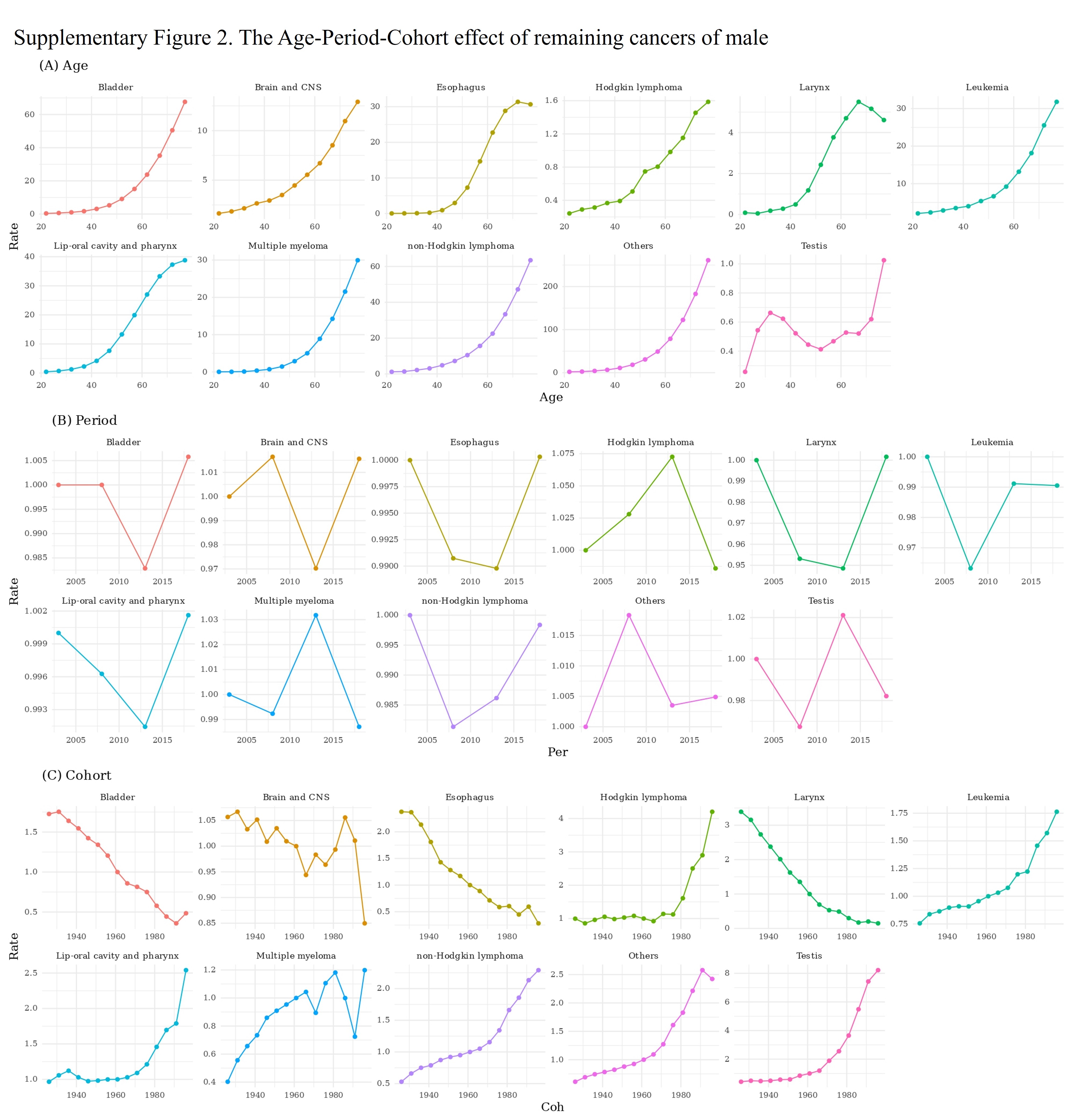

Supplement: Supplementary file 1 [file medicina-61-02179-s001.zip › Figure S2.jpg]

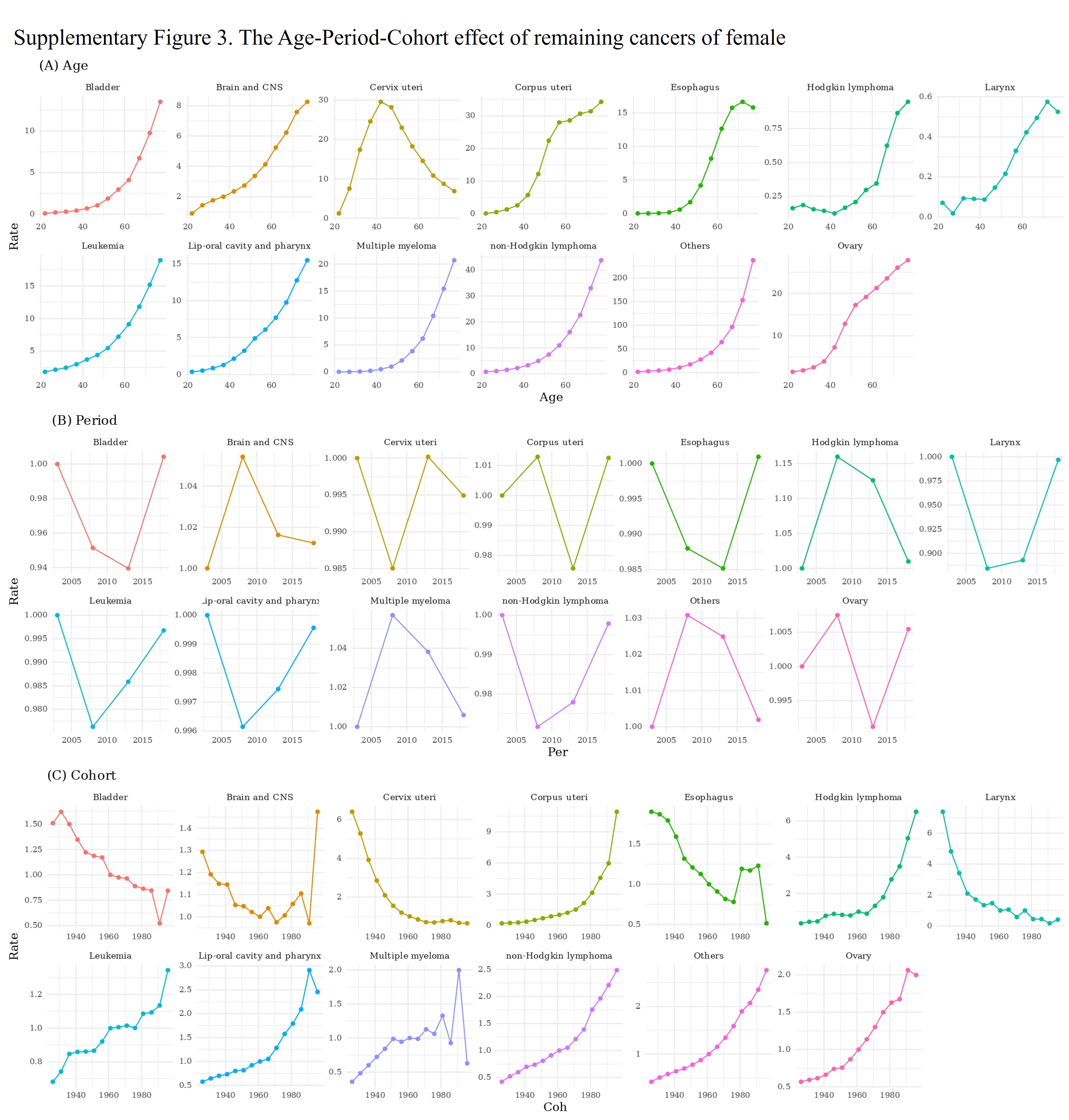

Supplement: Supplementary file 1 [file medicina-61-02179-s001.zip › Figure S3.jpg]

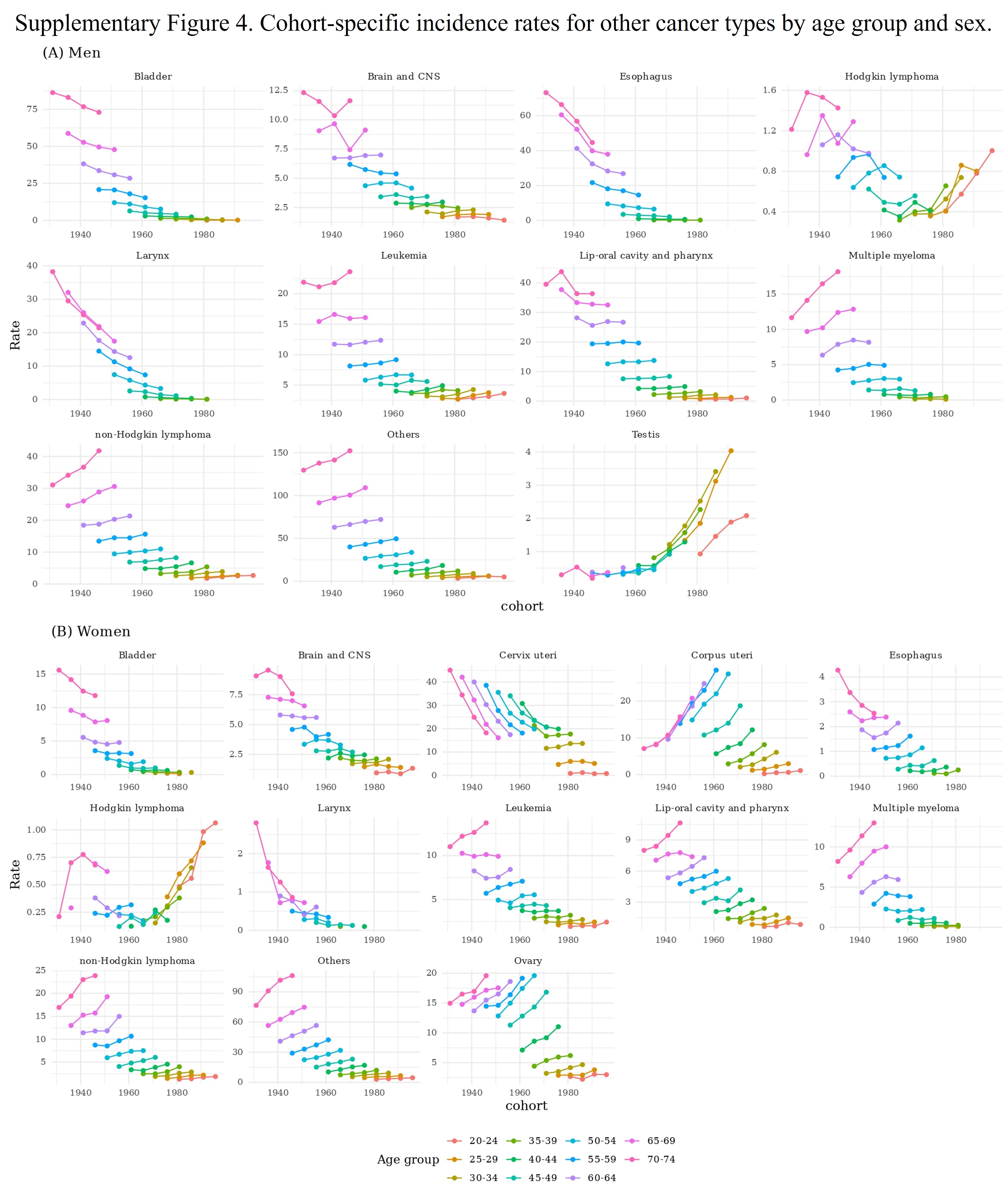

Supplement: Supplementary file 1 [file medicina-61-02179-s001.zip › Figure S4.jpg]
